# Supplementary figures and images for: Gaze dynamics prior to navigation support hierarchical planning
Source: PLoS One. 2026 Jun 11;21(6):e0351056. doi: 10.1371/journal.pone.0351056 (PMC13258153; doi:10.1371/journal.pone.0351056)

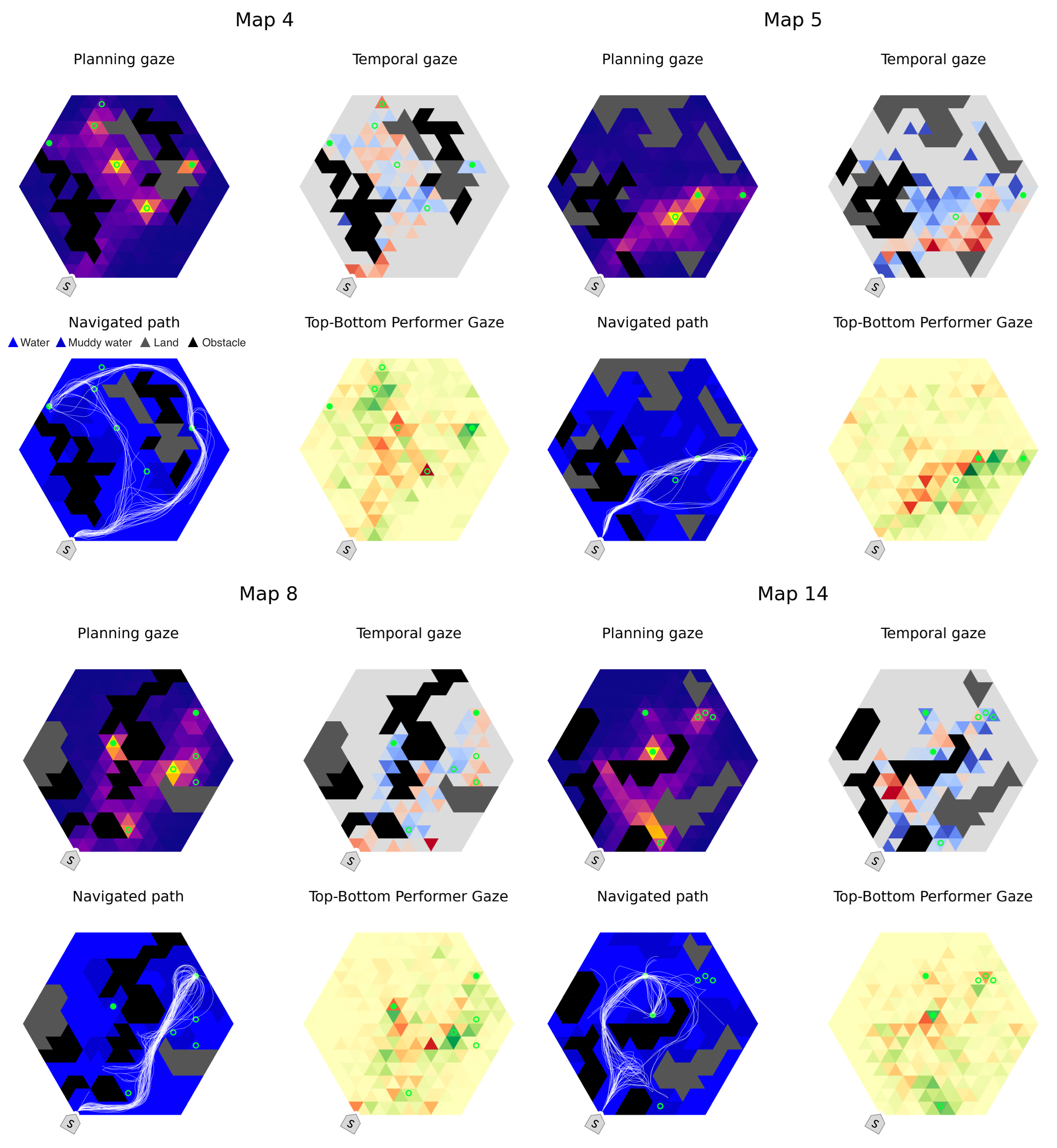

Supplement: S1 Fig — For each map, the following analyses are visualized: aggregated planning gaze (upper left), temporal gaze shift (upper right; blue tiles viewed earlier during planning, red later), path navigation trajectories (lower left), and performance-group differences in gaze density (lower right; green tiles show greater density among the top-performing group, and red tiles are those with greater density among the bottom-performing group.). (TIF) [file pone.0351056.s001.tif]

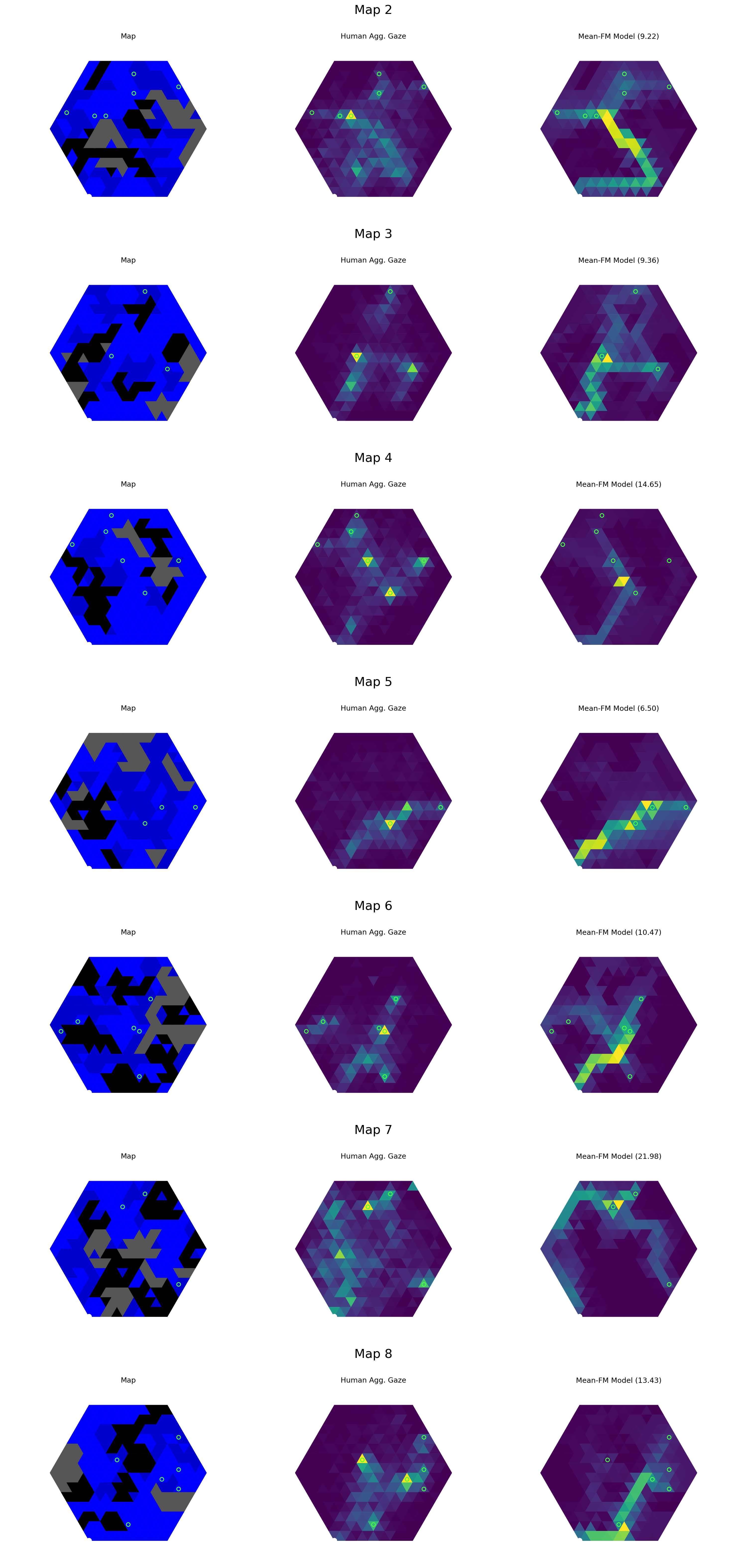

Supplement: S2 Fig — Number in title of FM model and agent gaze reports EMD from human aggregate gaze heatmap. Maps 2–8. (TIF) [file pone.0351056.s002.tif]

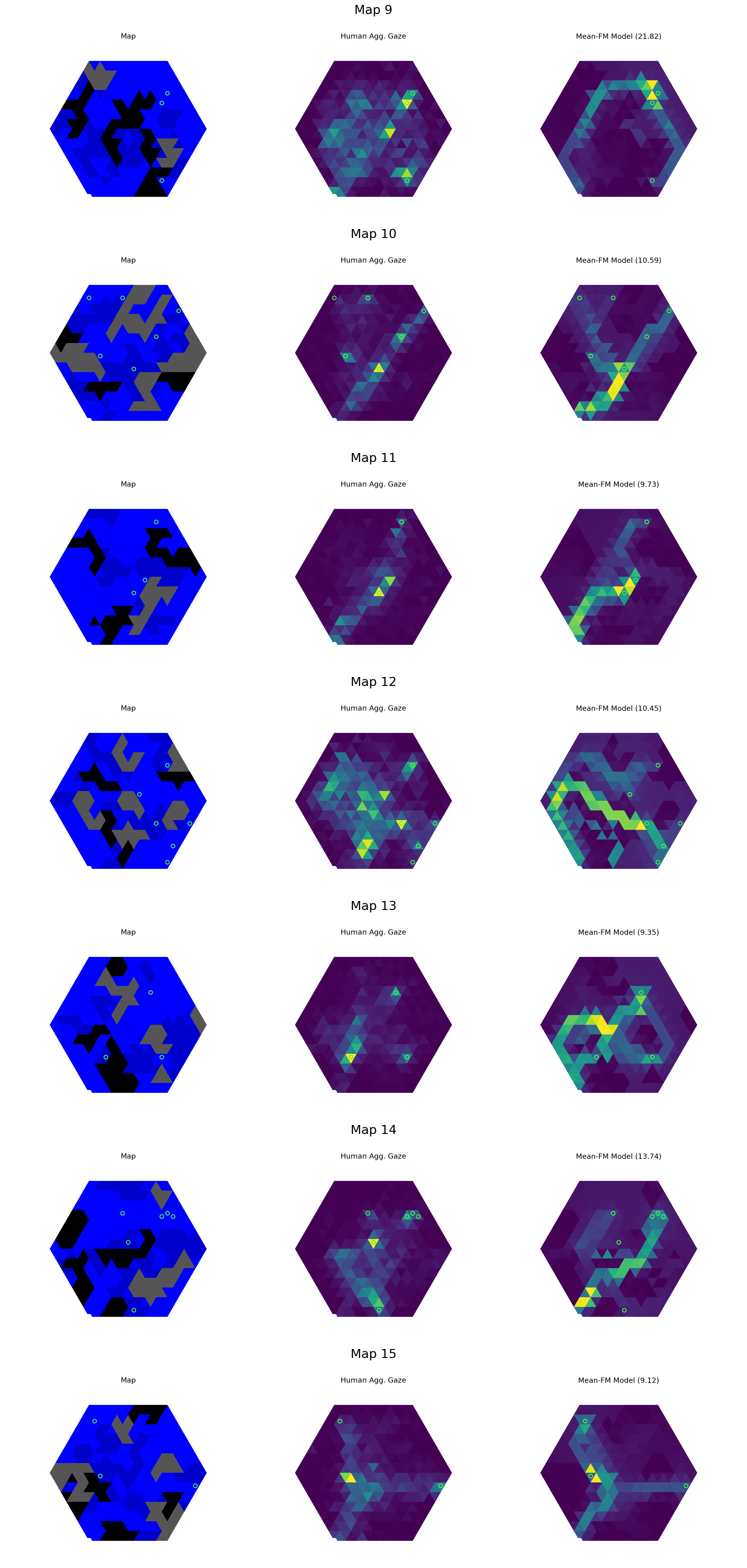

Supplement: S3 Fig — (TIF) [file pone.0351056.s003.tif]

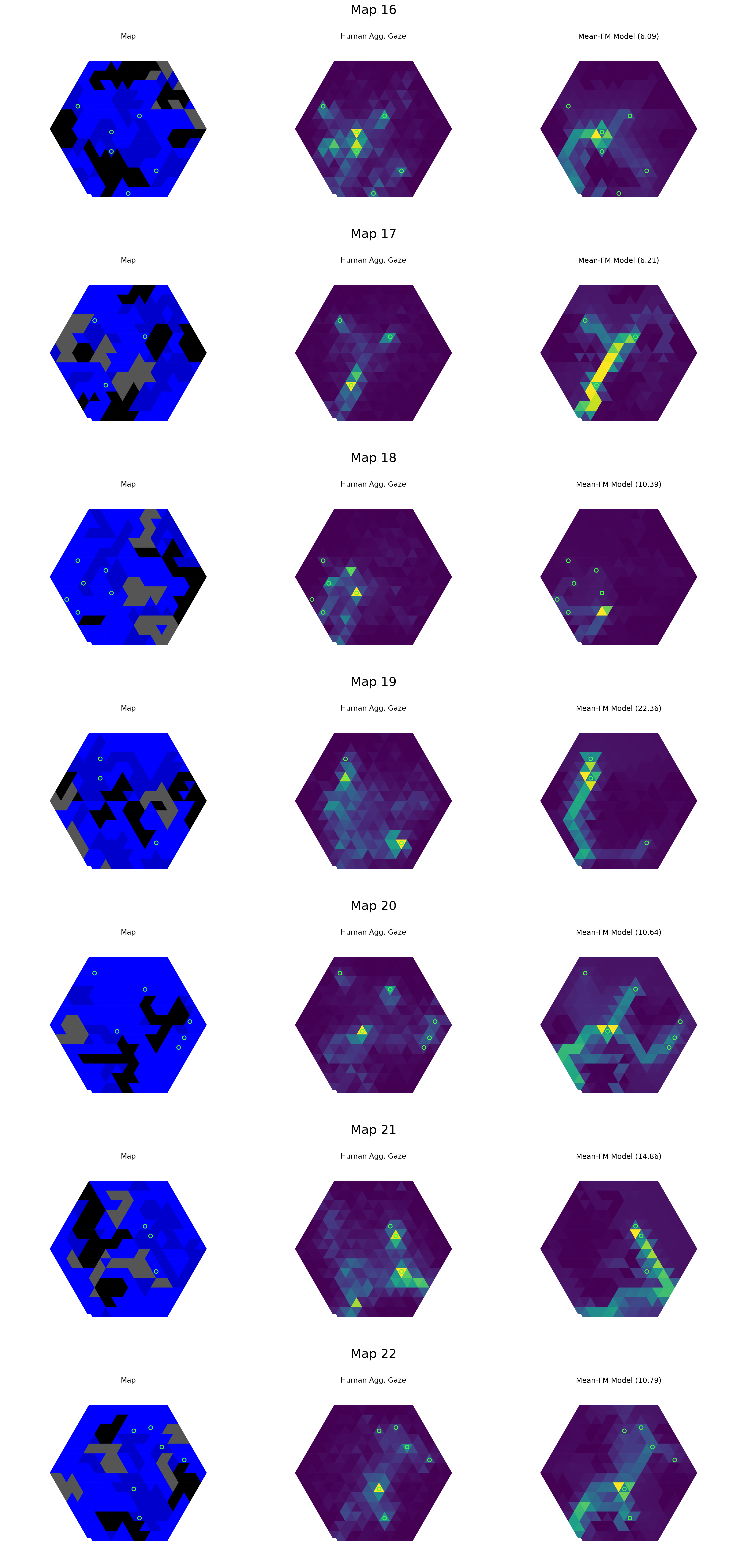

Supplement: S4 Fig — (TIF) [file pone.0351056.s004.tif]

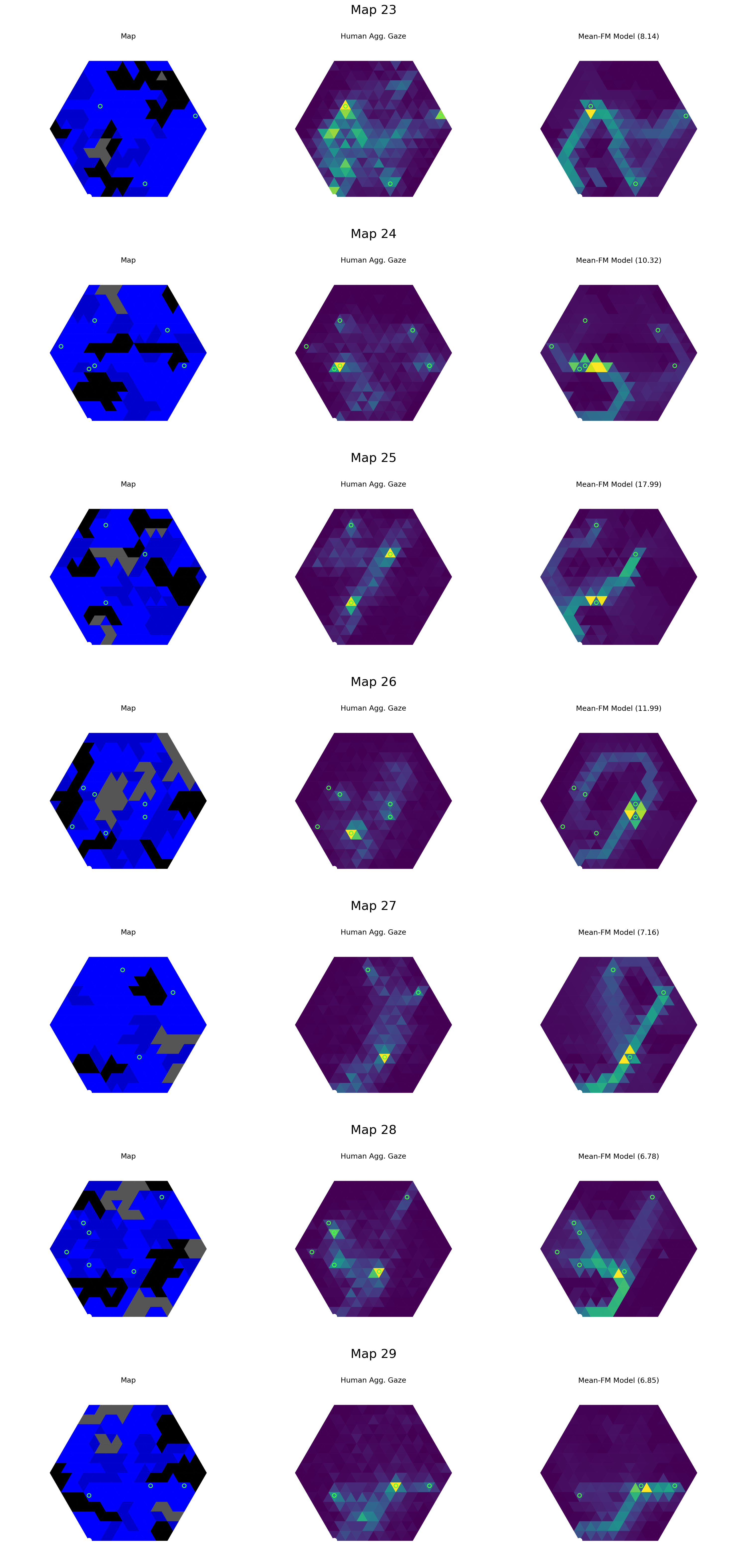

Supplement: S5 Fig — (TIF) [file pone.0351056.s005.tif]

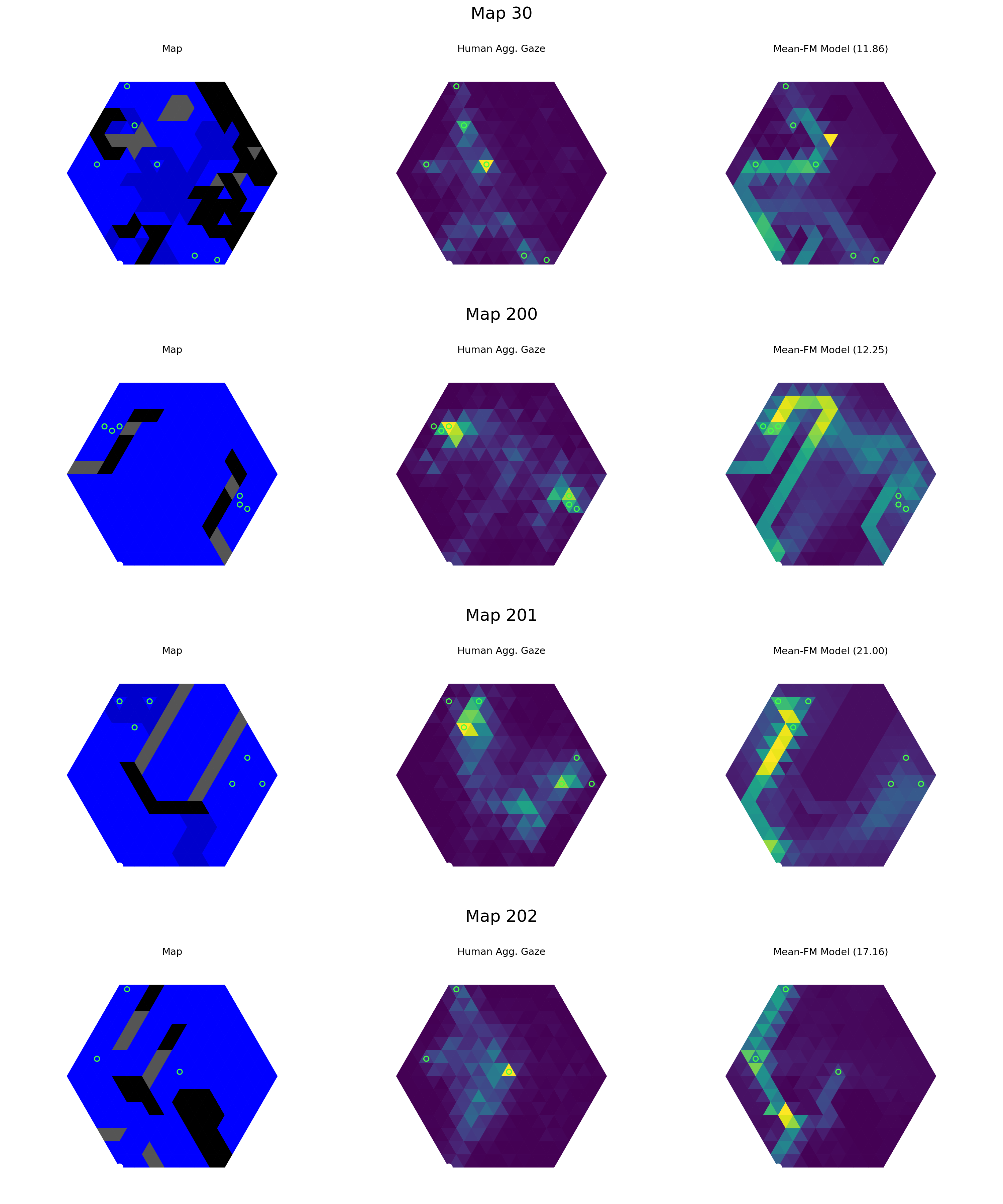

Supplement: S6 Fig — Map 30 and hand designed maps 200–202. (TIF) [file pone.0351056.s006.tif]

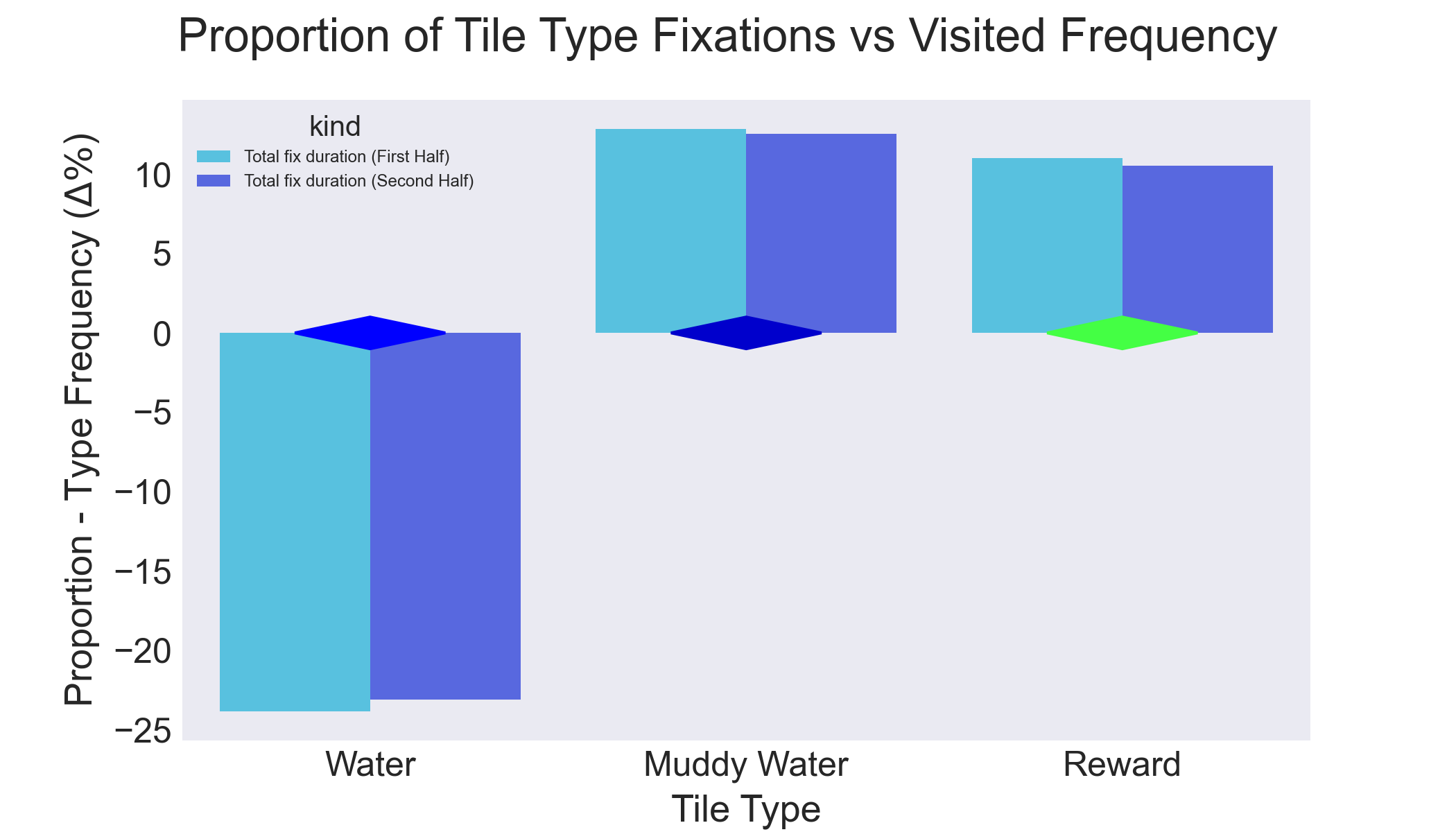

Supplement: S7 Fig — Similar analysis to 5 where type frequency is computed based on tile types visited, across participants, during navigation. (TIF) [file pone.0351056.s007.tif]
