# Supplementary material for: Gaze dynamics prior to navigation support hierarchical planning
Source: PLoS One. 2026 Jun 11;21(6):e0351056. doi: 10.1371/journal.pone.0351056 (PMC13258153; doi:10.1371/journal.pone.0351056)
Supplement: S1 Appendix — Map generation procedures. (PDF) [file pone.0351056.s012.pdf]

S1 Appendix - Map generation

Maps for the task were generated in a three step procedure: generate map pool, filter based on criteria, and finally, select an experimental sample. Below, we detail the procedure for each.

Procedurally generate map pool Map geometry was defined similarly to Zhu et al. but with boundaries defined by tile types, rather than walls drawn along tile edges. Specifically, a hexagonal grid composed of equilateral triangular tiles, and a side length of 7, formed the base map. Each tile could be set to one of four types: water (blue), muddy water (dark blue), land (gray), or obstacle (black). Land tiles were low altitude permitting vision, but not navigational access, while obstacle tiles were tall, and blocked visual access. To generate unique maps with variation in structure and connectivity, the algorithm below was followed:

```
Algorithm 1: Procedural map generation
Data: tileTypes[N] = WATER
;                                          /* Set all tiles initially to water */
Result: tileTypes[N], goalCaches[6]
n ← Uniform(10, 40) ;                      /* Choose n strokes to add */
i ← 0;
while i ≤ n do
    k ← Uniform(10, 40) ;                  /* Choose k stroke length */
    j ← 0;
    if i ≤ n/2 then
        type ← MWATER;
    else
        type ← choice(LAND, OBSTACLE);
    tileCursor = randomTile() while j ≤ k do
        tileTypes[tileCursor] = type;
        tileCursor = successors(tileCursor) ;   /* Successors is a list of adjacent
         tiles with prior visited tile removed */
        j ← j + 1;
    i ← i + 1;
goalCaches[] = {};
r ← 0;
while r ≤ 6 do
    goalCaches[r] = randomTile(WATER);
```

Filter pool To filter the pool of map candidates (defined by an assignment for each tile type, and an array of 6 goal cache locations), we confirmed that each one satisfies the following criteria:

1. No goal locations visible (line of sight) from origin
2. All goal locations reachable from origin
3. No obstacles adjacent to origin

Select experimental sample Finally, with the filtered pool of eligible map candidates, we selected 30 maps to compose the main experiment. We then randomly assigned half of the maps to each uncertainty group (low vs high). For both groups, we randomly chose 2 goals among the 6 goal caches to be present in the map. For the low uncertainty group, we then randomly removed 3 of the unused goal caches. The end result was a list of maps, where each contained 2 present goals, among either 3 or 6 possible goal cache locations.

Finally, we hand designed two practice maps with trivial geometric structures, to be used at the start of the experiment during the tutorial, as well as three maps testing other information geometries of interest. Map order was randomized for all participants, while ensuring that these latter three maps were always shown at the end of the experiment, to avoid any influence on the primary, procedurally generated maps.

Four sample maps from the main experiment can be seen in **S1 Fig** (lower-right figures show actual map as represented to participants during planning, with navigation trajectories overlayed).
