# Supplementary material for: Gaze dynamics prior to navigation support hierarchical planning
Source: PLoS One. 2026 Jun 11;21(6):e0351056. doi: 10.1371/journal.pone.0351056 (PMC13258153; doi:10.1371/journal.pone.0351056)
Supplement: S2 Appendix — Additional analyses on tile-type fixation frequency and proportion. (PDF) [file pone.0351056.s013.pdf]

S2 Appendix - Tile type visit frequency

While the aim of this study was the way gaze dynamics inform the exploration of many possible plans prior to execution, in light of Ho et al.'s findings suggesting that individuals pay little attention to task-irrelevant parts of the environment, we performed an additional analysis of tile-type fixation proportion that focuses on each individual's selected trajectory. The analysis was performed in parallel to the tile type analysis shown in **Fig 5**, however, here we compared fixation duration proportions with visit frequencies across all individuals' navigated routes (as opposed to the type distribution of the entire map). Results show a similar pattern of relative tile proportionality—less fixation on water, and more on deep water and reward tiles—which helps to validate that the source of the previously reported gaze distribution bias goes beyond overall map structure (see **S7 Fig**). We note that by restricting the comparison to tiles visited during navigation, the analysis necessarily excludes obstacles and land, which could not be navigated.
