# Supplementary material for: Gaze dynamics prior to navigation support hierarchical planning
Source: PLoS One. 2026 Jun 11;21(6):e0351056. doi: 10.1371/journal.pone.0351056 (PMC13258153; doi:10.1371/journal.pone.0351056)
Supplement: Attachment — Submitted filename: Response to Reviewers.pdf [file pone.0351056.s015.pdf]

Response to reviewers

Reviewer #1:

The paper asks whether (1) pre-navigation gaze reflects hierarchical planning and predicts future trajectories, (2) how subgoals are selected, and (3) which gaze patterns predict success. Participants perform a VR navigation task: they pilot a boat to collect a subset of goals from a larger set of potential cache locations. A location is confirmed as a goal only once it comes into line-of-sight, so planning must account for uncertainty and trade off maximizing goal collection with minimizing time/distance. The environment is a landscape whose features affect strategy: water and muddy water are navigable; land is not traversable; walls/obstacles block vision and movement. Each trial has three phases: (1) planning (15 s) by viewing a 2D map of the environment; (2) a brief transition; and (3) boat navigation in 3D (VR). Gaze transitions and fixations are recorded throughout, but analyses focus on the 15s planning phase. Results show robust relationships between planning-phase gaze and quantitative map features and task performance metrics, with clear signatures of a hierarchical, bidirectional planning process. These results hold across uncertainty conditions. Top and bottom performers differ: top performers show higher trajectory predictability from planning gaze and earlier attention to task-relevant regions. Finally, gaze concentrates on bottlenecks/subgoals (“critical tiles”) rather than arbitrary locations.

We thank the reviewer for the excellent summary and for the comments and suggestions which greatly helped us improve the paper. We describe below how we addressed the reviewer’s concerns, point by point.

Some notes:
The feature maps are visually appealing and intuitively convey task-relevant structure. However: figures would benefit from tile-specific labels (e.g. water, land) or reinforce them in the figure caption; the white start dot is very small and doesn’t immediately anchor the reader; each feature map should include a legend with its colormap range.

We thank the reviewer for this suggestion.

We updated the figures with improved legends and annotations. This includes a tile type legend at the top left of Figure 2, 3 and S1, a larger, clearer, start annotation identifying the participant's starting location on the map (Figure 2, 3 and S1, at the bottom left of each map render), and a colorbar showing the value range for feature maps (Figure 2). We also added a tile-type color key reminder in the caption of Figure 1, which is the first figure to show the top-down map layouts using the tile-type color schema.

To illustrate this, we are copying below the new figure 2 and its caption:

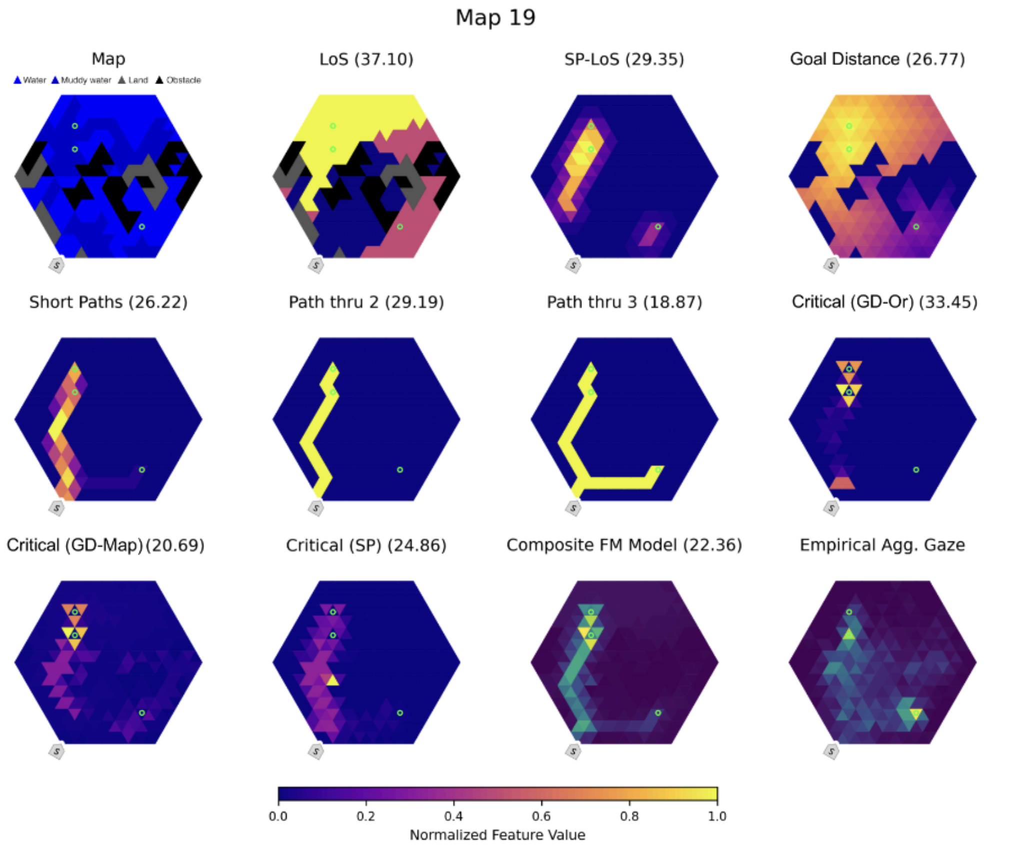

Fig 2. Sample feature and aggregated participant gaze for map 19. The first (top-left) figure shows map 19, in the format presented to participants. The subsequent 10 maps show different types of features, which comprise Line-of-Sight ("LoS"), Shortest-path Line-of-Sight ("SP-LoS"), distance to nearest goal ("Goal Distance"), Short Paths, Path through 2 goals, Path through 3 goals, Critical (via Goal-distance change at origin), Critical (via goal distance change map-wide), and Critical (via shortest path change). See Table 2 for details on each feature map. Note that the two critical feature maps identify bottleneck tiles such as small openings or possible shortcuts where inverting passability would enable or block a useful path. The mean of all feature maps is shown as the "Composite FM Model" in the second to last map. The last (bottom-right) figure shows the aggregated participants gaze for map 19. The numbers in parentheses above each map indicate the distributional distance between each feature-map and the aggregate gaze distribution using the 2D Euclidean earth distance (EMD).

Screenshot

It's a strength that the tile taxonomy maps onto real-world constraints (e.g., "muddy water" as a slowdown/crowding analogue). From a simple environment, the authors derive a rich set of planning-relevant features that plausibly generalize beyond the task.

Thanks for this comment.

It is interesting to observe how fixations shift between tile types across different planning phases. To strengthen this analysis, it would be helpful to include the distribution of each tile

type across the trajectory, to determine whether the observed differences in fixation patterns are driven by the structure of the environment or by participants' planning behavior.

Thanks for this useful suggestion.

We've performed an additional analysis of the tile type fixation durations compared with the distribution of tile types visited at navigation time. The below results have been added to the supporting information (in S2 Appendix). A result plot is added in a new supplementary figure (S7 Fig), reproduced below.

> "While the aim of this study was the way gaze dynamics inform the exploration of many possible plans prior to execution, in light of Ho et. al.'s findings suggesting that individuals pay little attention to task-irrelevant parts of the environment (Ho et. al., 2022), we performed an additional analysis of tile-type fixation proportion that focuses on each individual's selected trajectory. The analysis was performed in parallel to the tile type analysis shown in Fig 5, however, here we compared fixation duration proportions with visit frequencies across all individuals' navigated routes (as opposed to the type distribution of the entire map). Results show a similar pattern of relative tile proportionality---less fixation on water, and more on deep water and reward tiles---which helps to validate that the source of the previously reported gaze distribution bias goes beyond overall map structure (see S7 Fig). We note that by restricting the comparison to tiles visited during navigation, the analysis necessarily excludes obstacles and land, which could not be navigated."

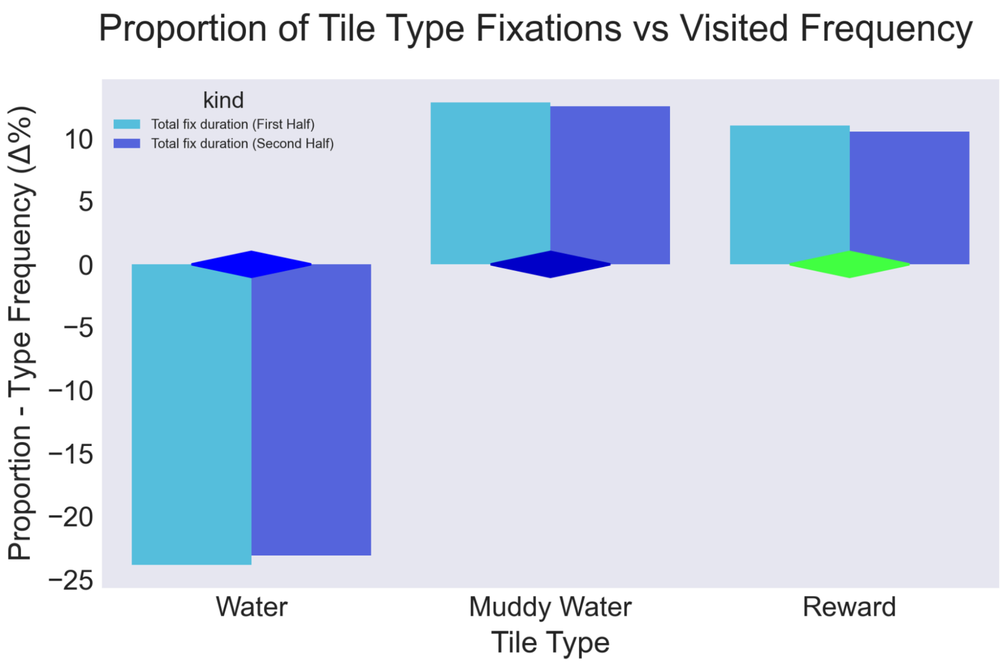

It would be helpful to see representative gaze trajectories for top vs. bottom performers to illustrate the most salient group differences (the current Supplementary Figure 1 points in this direction).

Thank you for this suggestion. To help address this, in the results section "Relation between path planning depth and performance," we've added a reference to aggregated gaze trajectory animations (viewable on our OSF project page), which are segmented by top and bottom performers, and show some of the gaze trajectory dynamics the reviews suggested elaboration on. We added a description of how to interpret these animations, and a sample observation from one map, consistent with cross-map statistical results discussed previously.

To aid interpretation (specially for immediate understanding of the 2D “info-graph” (Fig. 3)), consider adding small 3D environment snapshots highlighting key bottlenecks: cases where all goals are visible but the efficient path remains ambiguous because a wall forces a left/right corridor choice (memory-dependent uncertainty), and clear examples of “critical” tiles. Since critical tiles most strongly predict planning gaze, brief, plain-language definitions in the captions would help (e.g., a gate tile whose closure would block passage; a corridor mouth; an obstacle edge whose absence would reveal a goal).

We agree that seeing a 3D visualization of the participants' view of the maps and illustrating critical tiles and moments of uncertainty reduction would aid readers in understanding the planning and navigation dynamics, as well as the info-graphical map analyses. We've added a description of the "Participant POV" screen recording from within the VR display, which is added as a Supplementary Information file, and also linked on the OSF project site. In the revised manuscript, we noted the frame of this video when the participant encountered an information node of "no remaining uncertainty". We hope this will help readers to more deeply understand the info-graphical attributes, as well as the plan-dependent decisions central to our task.

Consider presenting how relevant critical tiles are for the optimal trajectory of each map to disentangle attention driven by task relevance from attention driven by map structure alone.

We are sorry for a less than clear exposition of the feature definition and analyses related to critical tiles. In our map feature analyses, we computed critical tiles in three different ways, and all approaches determined criticality quantitatively based on the direct impact of the tile's passability to map connectivity. This was not well explained, and we have updated the text in the caption of Table 2 to the following:

> "The three critical feature maps are designed following analyses in Zhu et. al. which quantify the importance of a tile to the navigation task by comparing connectivity when the tile is passable versus when it is blocked. In "Critical (GD-Origin)" we compare the impact on distance to goal at the origin. In "Critical (GD-Map)" we compare the total map-wide distance to the nearest goal. And finally, in "Critical (Shortest-Path)" we compare how much the shortest-path is impacted by each tile's passability."

We hope that this addresses the reviewer's concern.

It would be interesting to see how planning gaze compares to navigation gaze, i.e. how do offline planning and online planning gaze patterns differ.

While formal analyses of planning gaze were out of scope for this contribution, this is a very interesting direction for future work, and we have added this avenue to the revised manuscript in the "Limitations and future work" section. Navigation-time gaze data was captured in our study, and can be viewed in the "Aggregate Gaze Animations" videos on the OSF project page (https://osf.io/5tacn/), and a selection have been added as Supplementary Information, and referenced in the revised manuscript. These videos proceed in the order of each trial, and move through planning, transition, and navigation phases. Gaze is visualized for all 3 phases and can be seen in relation to the aggregate participant navigation trajectories as participants move through the map and view tiles visible from their present location.

As a minor point, there are a few grammatical errors and some figures not properly referenced in the text.

Thanks for noticing this. We have corrected a number of grammatical errors and ensured that all figures were properly referenced in the text according to the PLOS One style guide (the prior manuscript had used Figure instead of Fig in several places, and a non-standard supporting information sequencing).

I believe eye-tracking is a valuable tool for understanding internal cognitive processes in a non-invasive way, and this paper aligns well with that goal. It also has potential for growth and for the integration of more complex paradigms and behavioral recordings. I consider it a relevant and worthwhile contribution for publication after minor adjustments.

We thank the reviewer again for the useful guidance in improving the paper. We hope that the reviewer appreciates our revisions.

We've also annotated the ethics statement, which references the local IRB board that approved the study protocol, and clarified that both verbal and written consent were received prior to the experimental procedure.

Thank you again for the detailed review. We believe our revised manuscript has addressed most of the points raised, and hope that these changes have improved the strength of its contribution to the planning and decision-making literature.
