# Supplementary material for: Gaze dynamics prior to navigation support hierarchical planning
Source: PLoS One. 2026 Jun 11;21(6):e0351056. doi: 10.1371/journal.pone.0351056 (PMC13258153; doi:10.1371/journal.pone.0351056)
Supplement: Attachment — Submitted filename: Response_to_Reviewers_auresp_2.pdf [file pone.0351056.s016.pdf]

Response to reviewers

Reviewer #1:

Reviewer #1: I thank you for the careful consideration of the points raised by me in the first revision. Most of the points were accurately addressed. Figure 2 has greatly improved in clarity due to modified legends and annotations. For each map, animations were provided comparing top vs. bottom performers' gaze trajectories, which complement the reading in a meaningful way. I consider the new explanation of "critical tiles" in the description of Table 2 to be to the point and to clarify my initial concerns.

We are happy that the reviewer appreciated our revisions and we are thankful for the useful comments and suggestions.

A video of 3D navigation from the participant's POV was also provided, which improved the interpretation of Figure 3, and general interpretation of the 2D maps. I raise awareness for a timing mismatch in the text. The video is 2 minutes and 33 seconds long, and the authors point to the 29 seconds mark as referring to Map 6. However, the map at this timepoint corresponds to Map 2, with Map 6 only being shown from 53"-1'39".

Thanks for pointing this out, there were some differences between the video versions uploaded to the OSF project site, and the one submitted with the manuscript. We have now ensured that the supplementary video in both locations matches the textual description.

Regarding my previously raised point: "it would be helpful to include the distribution of each tile type across the trajectory, to determine whether the observed differences in fixation patterns are driven by the structure of the environment or by participants' planning behavior" – which was raised in Figure 5 and addressed in S2 Appendix, what I meant to ask was:

From what I see in Figure 4 (5th column), gaze trajectories are reward-to-origin, since distance from origin decreases after the initial jump. If a participant starts planning from the reward location, and there are in general more e.g. walls near the reward compared to near the origin, then the fixation duration of wall tiles would be higher in the first half of planning (closer to reward), compared to the second. If you normalize fixations by their frequency in the spatial segment covered by each half of the planning gaze trajectory in each map (not map frequency, nor visited frequency), this effect is balanced and results are more planning-relevant.

Nevertheless, the additional analysis comparing fixation duration proportions with visit frequencies across all individuals' navigated routes further validates the effect of higher attention taken to "muddy water" and "reward" as being driven by task relevancy, as seen in S7 Figure. And if we consider the spatial segment covered by planning gaze to be correlated to navigated routes, we can infer the results would be similar, with the only benefit of analysing these spatial segments being the inclusion of "land" and "wall" tile types. I thank you for this additional effort.

Overall, the paper is reading with more clarity and I believe it is ready for publication after minor adjustments.

Thanks for this suggestion and for the clarification. We agree that a limitation of our analysis could be an uneven distribution of tile types (e.g., “obstacles” and “water”) in the map, for example, closer to reward or origin locations. However, we are inclined to consider these sorts of asymmetries as standard features of naturalistic settings. We also note that including an additional analysis of tile-type fixation biases normalized by spatial frequency around true gaze targets in each half of the planning phase would require choosing an area of interest radius within which to normalize, thereby identifying disproportionate fixation choices (among nearby alternate options). It is unclear how best to select such a radius, and moreover, if broader map features (such as large areas of land) attract gaze, a regional normalization would fail to identify this dynamic. That said, we would like to include a paragraph that clearly identifies this issue, for the sake of the reader, and we added the following paragraph to the Discussion section:

A possible caveat with the interpretation of the results is that in “naturalistic” settings like ours, which include rich visual features and allow for free visual exploration, there are invariably some factors that cannot be fully controlled. For example, when considering the fixation duration proportions for different tile types, it is worth remembering that tile types might not be evenly distributed within the map (e.g., close to reward or origin locations) and even if they were, there is no guarantee that participants have the same prior probability to attend to them, as this depends on their particular choice of visual exploration and navigation strategies. For example, if participants first scan rewards and then the origin location, they might attend more frequently to the tile types that are more frequent in these zones. While these are intrinsic limitations of addressing rich ecological settings, these can be mitigated by more refined analyses or future experiments that explicitly disentangle these factors.

We hope that the reviewer agrees that this text helps the reader understand the possible constraints and caveats we should have in mind when interpreting results from experiments in which controlling all factors is challenging.

Thank you again for the detailed reviews.
