# Supplementary material for: Gaze dynamics prior to navigation support hierarchical planning
Source: PLoS One. 2026 Jun 11;21(6):e0351056. doi: 10.1371/journal.pone.0351056 (PMC13258153; doi:10.1371/journal.pone.0351056)
Supplement: Peer review history

# Peer review: Decision Letter 0

**DOI:** 10.1371/journal.pone.0351056.r001

**Authors:** Antoine Coutrot

5 Jan 2026

PONE-D-25-52975

Gaze dynamics prior to navigation support hierarchical planning

PLOS One

Dear Dr. Pezzulo,

Thank you for submitting your manuscript to PLOS ONE. After careful consideration, we feel that it has merit but does not fully meet PLOS ONE’s publication criteria as it currently stands. Therefore, we invite you to submit a revised version of the manuscript that addresses the points raised during the review process.

Please submit your revised manuscript by Feb 19 2026 11:59PM. If you will need more time than this to complete your revisions, please reply to this message or contact the journal office at plosone@plos.org. When you're ready to submit your revision, log on to https://www.editorialmanager.com/pone/ and select the 'Submissions Needing Revision' folder to locate your manuscript file.

Please include the following items when submitting your revised manuscript:

- A letter that responds to each point raised by the academic editor and reviewer(s). You should upload this letter as a separate file labeled 'Response to Reviewers'.
- A marked-up copy of your manuscript that highlights changes made to the original version. You should upload this as a separate file labeled 'Revised Manuscript with Track Changes'.
- An unmarked version of your revised paper without tracked changes. You should upload this as a separate file labeled 'Manuscript'.

-->If you would like to make changes to your financial disclosure, please include your updated statement in your cover letter. Guidelines for resubmitting your figure files are available below the reviewer comments at the end of this letter.

If applicable, we recommend that you deposit your laboratory protocols in protocols.io to enhance the reproducibility of your results. Protocols.io assigns your protocol its own identifier (DOI) so that it can be cited independently in the future. For instructions see: https://journals.plos.org/plosone/s/submission-guidelines#loc-laboratory-protocols. Additionally, PLOS ONE offers an option for publishing peer-reviewed Lab Protocol articles, which describe protocols hosted on protocols.io. Read more information on sharing protocols at https://plos.org/protocols?utm_medium=editorial-email&utm_source=authorletters&utm_campaign=protocols.

We look forward to receiving your revised manuscript.

Kind regards,

Antoine Coutrot

Academic Editor

PLOS One

Journal requirements:

When submitting your revision, we need you to address these additional requirements.

1. Please ensure that your manuscript meets PLOS ONE's style requirements, including those for file naming. The PLOS ONE style templates can be found at

https://journals.plos.org/plosone/s/file?id=wjVg/PLOSOne_formatting_sample_main_body.pdf and

https://journals.plos.org/plosone/s/file?id=ba62/PLOSOne_formatting_sample_title_authors_affiliations.pdf

2. Please include a complete copy of PLOS’ questionnaire on inclusivity in global research in your revised manuscript. Our policy for research in this area aims to improve transparency in the reporting of research performed outside of researchers’ own country or community. The policy applies to researchers who have travelled to a different country to conduct research, research with Indigenous populations or their lands, and research on cultural artefacts. The questionnaire can also be requested at the journal’s discretion for any other submissions, even if these conditions are not met.  Please find more information on the policy and a link to download a blank copy of the questionnaire here: https://journals.plos.org/plosone/s/best-practices-in-research-reporting. Please upload a completed version of your questionnaire as Supporting Information when you resubmit your manuscript.

3. We note that the grant information you provided in the ‘Funding Information’ and ‘Financial Disclosure’ sections do not match.

When you resubmit, please ensure that you provide the correct grant numbers for the awards you received for your study in the ‘Funding Information’ section.

4. Please include your full ethics statement in the ‘Methods’ section of your manuscript file. In your statement, please include the full name of the IRB or ethics committee who approved or waived your study, as well as whether or not you obtained informed written or verbal consent. If consent was waived for your study, please include this information in your statement as well.

If the reviewer comments include a recommendation to cite specific previously published works, please review and evaluate these publications to determine whether they are relevant and should be cited. There is no requirement to cite these works unless the editor has indicated otherwise.

Please review your reference list to ensure that it is complete and correct. If you have cited papers that have been retracted, please include the rationale for doing so in the manuscript text, or remove these references and replace them with relevant current references. Any changes to the reference list should be mentioned in the rebuttal letter that accompanies your revised manuscript. If you need to cite a retracted article, indicate the article’s retracted status in the References list and also include a citation and full reference for the retraction notice.

[Note: HTML markup is below. Please do not edit.]

Reviewers' comments:

Reviewer's Responses to Questions

**Comments to the Author**

1. Is the manuscript technically sound, and do the data support the conclusions?

The manuscript must describe a technically sound piece of scientific research with data that supports the conclusions. Experiments must have been conducted rigorously, with appropriate controls, replication, and sample sizes. The conclusions must be drawn appropriately based on the data presented.

Reviewer #1: Yes

**********

2. Has the statistical analysis been performed appropriately and rigorously?

Reviewer #1: Yes

**********

3. Have the authors made all data underlying the findings in their manuscript fully available?

The PLOS Data policy requires authors to make all data underlying the findings described in their manuscript fully available without restriction, with rare exception (please refer to the Data Availability Statement in the manuscript PDF file). The data should be provided as part of the manuscript or its supporting information, or deposited to a public repository. For example, in addition to summary statistics, the data points behind means, medians and variance measures should be available. If there are restrictions on publicly sharing data—e.g. participant privacy or use of data from a third party—those must be specified.

Reviewer #1: Yes

**********

4. Is the manuscript presented in an intelligible fashion and written in standard English?

PLOS ONE does not copyedit accepted manuscripts, so the language in submitted articles must be clear, correct, and unambiguous. Any typographical or grammatical errors should be corrected at revision, so please note any specific errors here.

Reviewer #1: Yes

**********

5. Review Comments to the Author

Please use the space provided to explain your answers to the questions above. You may also include additional comments for the author, including concerns about dual publication, research ethics, or publication ethics. (Please upload your review as an attachment if it exceeds 20,000 characters)

Reviewer #1: The paper asks whether (1) pre-navigation gaze reflects hierarchical planning and predicts future trajectories, (2) how subgoals are selected, and (3) which gaze patterns predict success. Participants perform a VR navigation task: they pilot a boat to collect a subset of goals from a larger set of potential cache locations. A location is confirmed as a goal only once it comes into line-of-sight, so planning must account for uncertainty and trade off maximizing goal collection with minimizing time/distance. The environment is a landscape whose features affect strategy: water and muddy water are navigable; land is not traversable; walls/obstacles block vision and movement.

Each trial has three phases: (1) planning (15 s) by viewing a 2D map of the environment; (2) a brief transition; and (3) boat navigation in 3D (VR). Gaze transitions and fixations are recorded throughout, but analyses focus on the 15s planning phase.

Results show robust relationships between planning-phase gaze and quantitative map features and task performance metrics, with clear signatures of a hierarchical, bidirectional planning process. These results hold across uncertainty conditions. Top and bottom performers differ: top performers show higher trajectory predictability from planning gaze and earlier attention to task-relevant regions. Finally, gaze concentrates on bottlenecks/subgoals (“critical tiles”) rather than arbitrary locations.

Some notes:

The feature maps are visually appealing and intuitively convey task-relevant structure. However:

figures would benefit from tile-specific labels (e.g. water, land) or reinforce them in the figure caption;

the white start dot is very small and doesn’t immediately anchor the reader;

each feature map should include a legend with its colormap range.

It’s a strength that the tile taxonomy maps onto real-world constraints (e.g., “muddy water” as a slowdown/crowding analogue). From a simple environment, the authors derive a rich set of planning-relevant features that plausibly generalize beyond the task.

It is interesting to observe how fixations shift between tile types across different planning phases. To strengthen this analysis, it would be helpful to include the distribution of each tile type across the trajectory, to determine whether the observed differences in fixation patterns are driven by the structure of the environment or by participants’ planning behavior.

It would be helpful to see representative gaze trajectories for top vs. bottom performers to illustrate the most salient group differences (the current Supplementary Figure 1 points in this direction).

To aid interpretation (specially for immediate understanding of the 2D “info-graph” (Fig. 3)), consider adding small 3D environment snapshots highlighting key bottlenecks: cases where all goals are visible but the efficient path remains ambiguous because a wall forces a left/right corridor choice (memory-dependent uncertainty), and clear examples of “critical” tiles.

Since critical tiles most strongly predict planning gaze, brief, plain-language definitions in the captions would help (e.g., a gate tile whose closure would block passage; a corridor mouth; an obstacle edge whose absence would reveal a goal).

Consider presenting how relevant critical tiles are for the optimal trajectory of each map to disentangle attention driven by task relevance from attention driven by map structure alone.

It would be interesting to see how planning gaze compares to navigation gaze, i.e. how do offline planning and online planning gaze patterns differ.

Overall, the exposition of several concepts could be tightened, but the map visualizations are compelling, and the targeting of different features across the planned trajectory is well motivated. None of these presentation issues alter the core contribution: planning-time gaze provides a sensitive window onto hierarchical subgoaling in navigation, and attention concentrates on the true hinge points of the environment. As a minor point, there are a few grammatical errors and some figures not properly referenced in the text. I believe eye-tracking is a valuable tool for understanding internal cognitive processes in a non-invasive way, and this paper aligns well with that goal. It also has potential for growth and for the integration of more complex paradigms and behavioral recordings. I consider it a relevant and worthwhile contribution for publication after minor adjustments.

**********

6. PLOS authors have the option to publish the peer review history of their article (what does this mean?). If published, this will include your full peer review and any attached files.

If you choose “no”, your identity will remain anonymous but your review may still be made public.

**Do you want your identity to be public for this peer review?**   For information about this choice, including consent withdrawal, please see our Privacy Policy.

Reviewer #1: No

**********

[NOTE: If reviewer comments were submitted as an attachment file, they will be attached to this email and accessible via the submission site. Please log into your account, locate the manuscript record, and check for the action link "View Attachments". If this link does not appear, there are no attachment files.]

To ensure your figures meet our technical requirements, please review our figure guidelines: https://journals.plos.org/plosone/s/figures

You may also use PLOS’s free figure tool, NAAS, to help you prepare publication quality figures: https://journals.plos.org/plosone/s/figures#loc-tools-for-figure-preparation.

NAAS will assess whether your figures meet our technical requirements by comparing each figure against our figure specifications.

# Author comment: Author response to Decision Letter 1

**DOI:** 10.1371/journal.pone.0351056.r002

13 Feb 2026

Please find our response to reviewers letter in the attached document list.

Attachment Submitted filename: Response to Reviewers.pdf

# Peer review: Decision Letter 1

**DOI:** 10.1371/journal.pone.0351056.r003

**Authors:** Antoine Coutrot

23 Mar 2026

PONE-D-25-52975R1

Gaze dynamics prior to navigation support hierarchical planning

PLOS One

Dear Dr. Pezzulo,

Thank you for submitting your revised manuscript to PLOS ONE. Based on the reviewers' comments, we believe your manuscript is almost ready for publication, pending minor changes. Therefore, we invite you to submit a revised version of the manuscript that addresses the points raised during the review process.

Please submit your revised manuscript by May 07 2026 11:59PM. If you will need more time than this to complete your revisions, please reply to this message or contact the journal office at plosone@plos.org. When you're ready to submit your revision, log on to https://www.editorialmanager.com/pone/ and select the 'Submissions Needing Revision' folder to locate your manuscript file.

Please include the following items when submitting your revised manuscript:

- A letter that responds to each point raised by the academic editor and reviewer(s). You should upload this letter as a separate file labeled 'Response to Reviewers'.
- A marked-up copy of your manuscript that highlights changes made to the original version. You should upload this as a separate file labeled 'Revised Manuscript with Track Changes'.
- An unmarked version of your revised paper without tracked changes. You should upload this as a separate file labeled 'Manuscript'.

-->If you would like to make changes to your financial disclosure, please include your updated statement in your cover letter. Guidelines for resubmitting your figure files are available below the reviewer comments at the end of this letter.

If applicable, we recommend that you deposit your laboratory protocols in protocols.io to enhance the reproducibility of your results. Protocols.io assigns your protocol its own identifier (DOI) so that it can be cited independently in the future. For instructions see: https://journals.plos.org/plosone/s/submission-guidelines#loc-laboratory-protocols. Additionally, PLOS ONE offers an option for publishing peer-reviewed Lab Protocol articles, which describe protocols hosted on protocols.io. Read more information on sharing protocols at https://plos.org/protocols?utm_medium=editorial-email&utm_source=authorletters&utm_campaign=protocols.

We look forward to receiving your revised manuscript.

Kind regards,

Antoine Coutrot

Academic Editor

PLOS One

Journal Requirements:

If the reviewer comments include a recommendation to cite specific previously published works, please review and evaluate these publications to determine whether they are relevant and should be cited. There is no requirement to cite these works unless the editor has indicated otherwise.

Please review your reference list to ensure that it is complete and correct. If you have cited papers that have been retracted, please include the rationale for doing so in the manuscript text, or remove these references and replace them with relevant current references. Any changes to the reference list should be mentioned in the rebuttal letter that accompanies your revised manuscript. If you need to cite a retracted article, indicate the article’s retracted status in the References list and also include a citation and full reference for the retraction notice.

[Note: HTML markup is below. Please do not edit.]

Reviewers' comments:

Reviewer's Responses to Questions

**Comments to the Author**

1. If the authors have adequately addressed your comments raised in a previous round of review and you feel that this manuscript is now acceptable for publication, you may indicate that here to bypass the “Comments to the Author” section, enter your conflict of interest statement in the “Confidential to Editor” section, and submit your "Accept" recommendation.

Reviewer #1: (No Response)

**********

2. Is the manuscript technically sound, and do the data support the conclusions?

The manuscript must describe a technically sound piece of scientific research with data that supports the conclusions. Experiments must have been conducted rigorously, with appropriate controls, replication, and sample sizes. The conclusions must be drawn appropriately based on the data presented.

Reviewer #1: Yes

**********

3. Has the statistical analysis been performed appropriately and rigorously?

Reviewer #1: Yes

**********

4. Have the authors made all data underlying the findings in their manuscript fully available?

The PLOS Data policy requires authors to make all data underlying the findings described in their manuscript fully available without restriction, with rare exception (please refer to the Data Availability Statement in the manuscript PDF file). The data should be provided as part of the manuscript or its supporting information, or deposited to a public repository. For example, in addition to summary statistics, the data points behind means, medians and variance measures should be available. If there are restrictions on publicly sharing data—e.g. participant privacy or use of data from a third party—those must be specified.

Reviewer #1: Yes

**********

5. Is the manuscript presented in an intelligible fashion and written in standard English?

PLOS ONE does not copyedit accepted manuscripts, so the language in submitted articles must be clear, correct, and unambiguous. Any typographical or grammatical errors should be corrected at revision, so please note any specific errors here.

Reviewer #1: Yes

**********

6. Review Comments to the Author

Please use the space provided to explain your answers to the questions above. You may also include additional comments for the author, including concerns about dual publication, research ethics, or publication ethics. (Please upload your review as an attachment if it exceeds 20,000 characters)

Reviewer #1: I thank you for the careful consideration of the points raised by me in the first revision. Most of the points were accurately addressed. Figure 2 has greatly improved in clarity due to modified legends and annotations. For each map, animations were provided comparing top vs. bottom performers’ gaze trajectories, which complement the reading in a meaningful way. I consider the new explanation of “critical tiles” in the description of Table 2 to be to the point and to clarify my initial concerns.

A video of 3D navigation from the participant’s POV was also provided, which improved the interpretation of Figure 3, and general interpretation of the 2D maps. I raise awareness for a timing mismatch in the text. The video is 2 minutes and 33 seconds long, and the authors point to the 29 seconds mark as referring to Map 6. However, the map at this timepoint corresponds to Map 2, with Map 6 only being shown from 53”-1’39”.

Regarding my previously raised point: “it would be helpful to include the distribution of each tile type across the trajectory, to determine whether the observed differences in fixation patterns are driven by the structure of the environment or by participants’ planning behavior” – which was raised in Figure 5 and addressed in S2 Appendix, what I meant to ask was:

From what I see in Figure 4 (5th column), gaze trajectories are reward-to-origin, since distance from origin decreases after the initial jump. If a participant starts planning from the reward location, and there are in general more e.g. walls near the reward compared to near the origin, then the fixation duration of wall tiles would be higher in the first half of planning (closer to reward), compared to the second. If you normalize fixations by their frequency in the spatial segment covered by each half of the planning gaze trajectory in each map (not map frequency, nor visited frequency), this effect is balanced and results are more planning-relevant.

Nevertheless, the additional analysis comparing fixation duration proportions with visit frequencies across all individuals' navigated routes further validates the effect of higher attention taken to “muddy water” and “reward” as being driven by task relevancy, as seen in S7 Figure. And if we consider the spatial segment covered by planning gaze to be correlated to navigated routes, we can infer the results would be similar, with the only benefit of analysing these spatial segments being the inclusion of “land” and “wall” tile types. I thank you for this additional effort.

Overall, the paper is reading with more clarity and I believe it is ready for publication after minor adjustments.

**********

7. PLOS authors have the option to publish the peer review history of their article (what does this mean?). If published, this will include your full peer review and any attached files.

If you choose “no”, your identity will remain anonymous but your review may still be made public.

**Do you want your identity to be public for this peer review?**   For information about this choice, including consent withdrawal, please see our Privacy Policy.

Reviewer #1: No

**********

[NOTE: If reviewer comments were submitted as an attachment file, they will be attached to this email and accessible via the submission site. Please log into your account, locate the manuscript record, and check for the action link "View Attachments". If this link does not appear, there are no attachment files.]

To ensure your figures meet our technical requirements, please review our figure guidelines: https://journals.plos.org/plosone/s/figures

You may also use PLOS’s free figure tool, NAAS, to help you prepare publication quality figures: https://journals.plos.org/plosone/s/figures#loc-tools-for-figure-preparation.

NAAS will assess whether your figures meet our technical requirements by comparing each figure against our figure specifications.

# Author comment: Author response to Decision Letter 2

**DOI:** 10.1371/journal.pone.0351056.r004

27 Apr 2026

We've made revisions as requested by the latest review comments. These have been uploaded with tracked changes and as a final manuscripts, along with an updated response to reviewers letter.

Attachment Submitted filename: Response_to_Reviewers_auresp_2.pdf

# Editor report: Decision Letter 2

**DOI:** 10.1371/journal.pone.0351056.r005

**Authors:** Antoine Coutrot

21 May 2026

Gaze dynamics prior to navigation support hierarchical planning

PONE-D-25-52975R2

Dear Dr. Pezzulo,

We’re pleased to inform you that your manuscript has been judged scientifically suitable for publication and will be formally accepted for publication once it meets all outstanding technical requirements.

Within one week, you’ll receive an e-mail detailing the required amendments. When these have been addressed, you’ll receive a formal acceptance letter and your manuscript will be scheduled for publication.

An invoice will be generated when your article is formally accepted. Please note, if your institution has a publishing partnership with PLOS and your article meets the relevant criteria, all or part of your publication costs will be covered. Please make sure your user information is up-to-date by logging into Editorial Manager at Editorial Manager® and clicking the ‘Update My Information' link at the top of the page. For questions related to billing, please contact billing support.

If your institution or institutions have a press office, please notify them about your upcoming paper to help maximize its impact. If they’ll be preparing press materials, please inform our press team as soon as possible -- no later than 48 hours after receiving the formal acceptance. Your manuscript will remain under strict press embargo until 2 pm Eastern Time on the date of publication. For more information, please contact onepress@plos.org.

Kind regards,

Antoine Coutrot

Academic Editor

PLOS One

Additional Editor Comments (optional):

Reviewers' comments:

# Editor report: Acceptance letter

**DOI:** 10.1371/journal.pone.0351056.r006

**Authors:** Antoine Coutrot

PONE-D-25-52975R2

PLOS One

Dear Dr. Pezzulo,

I'm pleased to inform you that your manuscript has been deemed suitable for publication in PLOS One. Congratulations! Your manuscript is now being handed over to our production team.

At this stage, our production department will prepare your paper for publication. This includes ensuring the following:

* All references, tables, and figures are properly cited

* All relevant supporting information is included in the manuscript submission,

* There are no issues that prevent the paper from being properly typeset

You will receive further instructions from the production team, including instructions on how to review your proof when it is ready. Please keep in mind that we are working through a large volume of accepted articles, so please give us a few days to review your paper and let you know the next and final steps.

Lastly, if your institution or institutions have a press office, please let them know about your upcoming paper now to help maximize its impact. If they'll be preparing press materials, please inform our press team within the next 48 hours. Your manuscript will remain under strict press embargo until 2 pm Eastern Time on the date of publication. For more information, please contact onepress@plos.org.

You will receive an invoice from PLOS for your publication fee after your manuscript has reached the completed accept phase. If you receive an email requesting payment before acceptance or for any other service, this may be a phishing scheme. Learn how to identify phishing emails and protect your accounts at https://explore.plos.org/phishing.

If we can help with anything else, please email us at customercare@plos.org.

Thank you for submitting your work to PLOS ONE and supporting open access.

Kind regards,

PLOS ONE Editorial Office Staff

on behalf of

Dr. Antoine Coutrot

Academic Editor

PLOS One
